# The immunogenicity, safety, and consistency of an Indonesia combined DTP-HB-Hib vaccine in expanded program on immunization schedule

**DOI:** 10.1186/s12887-015-0525-2

**Published:** 2015-12-19

**Authors:** Kusnandi Rusmil, Hartono Gunardi, Eddy Fadlyana, Soedjatmiko, Meita Dhamayanti, Rini Sekartini, Hindra Irawan Satari, Nelly Amalia Risan, Dwi Prasetio, Rodman Tarigan, Reni Garheni, Mia Milanti, Sri Rezeki Hadinegoro, Suganda Tanuwidjaja, Novilia Sjafri Bachtiar, Rini Mulia Sari

**Affiliations:** Child Health Department, Faculty of Medicine, Padjadjaran University / Dr. Hasan Sadikin Hospital, Bandung, Indonesia; Child Health Department, Faculty of Medicine, University of Indonesia / Dr. Cipto Mangunkusumo Hospital, Jakarta, Indonesia; PT. Bio Farma, Bandung, Indonesia

**Keywords:** Combined DTP-HB-Hib vaccine, Infants, Primary vaccination, EPI

## Abstract

**Background:**

WHO recommended incorporation of *Haemophilus influenzae* type b (Hib) vaccination into immunization program. Indonesia would adopt Hib as a National Immunization Program in 2013. We aimed at analyzing immunogenicity, safety, and consistency of a new combined DTP-HB-Hib (diphtheria-tetanus-pertussis-Hepatitis B-Haemophilus influenza B) vaccine.

**Methods:**

A prospective, randomized, double blind, multicenter, phase III study of Bio Farma DTP-HB-Hib vaccine conducted in Jakarta and Bandung, August 2012 - January 2013.

Subjects were divided into three groups with different batch number. Healthy infants 6–11 weeks of age at enrollment were immunized with 3 doses of DTP-HB-Hib vaccine with interval of 4 weeks, after birth dose of hepatitis B vaccine. Blood samples obtained prior to vaccination and 28 days after the third dose. Safety measures recorded until 28 days after each dose.

**Results:**

Of 600 subjects, 575 (96 %) completed study protocol. After 3 doses, 100.0 and 96.0 % had anti-PRP concentration ≥0.15 and ≥1.0 μg/ml. Anti-diphtheria and anti-tetanus concentration ≥0.01 IU/ml detected in 99.7 and 100.0 %; while concentration ≥0.1 IU/ml achieved in 84.0 and 97.4 %. Protective anti-HBs found in 99.3 %. The pertussis vaccine response rate was 84.9 %.

None Serious Adverse events (SAEs) considered related to study vaccine or procedure.

**Conclusions:**

The 3-dose of DTP-HB-Hib was immunogenic, well tolerated and suitable for replacement of licensed-equivalent vaccines based on immunologic and safety profiles.

**Trial registration:**

NCT01986335 – October 30^th^ 2013.

## Background

*Haemophilus Influenza* type b is the leading cause of childhood bacterial pneumonia, meningitis, and other serious infections [1, 2]. In Indonesia, pneumonia and meningitis cause an estimated 15.5 and 8.8 % of all deaths recorded in under-five children, respectively [3]. Studies have reported that the majority of Hib-related pneumonia and meningitis occur in the first year of life [4, 5].

WHO has recommended the world wide incorporation of Hib vaccination into all routine infant immunization programs after 6 weeks of age, preferably as a diphtheria-tetanus-pertussis (DTP) based combination allowing rapid integration into existing DTP vaccination schedules [2, 6]. DTP-HB vaccine was licensed in Indonesia in 2004 and has been routinely given to infants at 2, 3, 4 months of age. Phase I and II study of DTP-HB-Hib vaccine showed that DTP-HB vaccine was subsequently shown to be immunogenic and well tolerated when mixed with Hib vaccine and administered as a single injection (DTP-HB-Hib) and already routinely used in many countries in the world [7–9].

Meanwhile, introduction of such combined vaccines in other middle and low income countries has been followed by serious concerns about safety and adverse events following immunization (AEFI). In 2008, the Advisory Committee on Communicable Diseases (ACCD) in Sri Lanka recommended to suspend the introduction of DPT-Hepatitis B-Hib vaccine, following several cases of hypotonic hyporesponsive episodes (HHE) which resulted in five deaths and decided to reintroduce the vaccine after both the Committee and WHO (World Health Organization) had found no conclusive evidence that the vaccine caused the deaths in their investigations [10]. In some developing countries, serious AEFI cases occurred, including Bhutan, India, and Vietnam from 2009 to 2013 [11].

The objective of this study is to evaluate the immunogenicity, safety, and consistency of lots of a new combined Bio Farma DTP-HB-Hib vaccine, when used as the primary vaccination of Indonesian infants according to EPI schedule at 6, 10, and 14 weeks of age, after a birth dose of hepatitis B vaccine, as recommended by WHO.

## Methods

### Study design and population

This was a prospective, randomized, double blind, multicenter, phase III study of combined DTP-HB-Hib vaccine. The study was conducted at 6 primary health centers in Jakarta and Bandung from August 2012 through January 2013 and was approved by Health Research Ethics Committee Faculty of Medicine University of Indonesia – Dr. Cipto Mangunkusumo Hospital and Health Research Ethics Committee Faculty of Medicine Padjajaran University – Dr. Hasan Sadikin Hospital. Parents or legal guardian of all subjects provided written informed consent before enrollment. The study was conducted in accordance with the Declaration of Helsinki and Good Clinical Practice guidelines.

The study population comprised of healthy infants who were 6–11 weeks of age at enrollment, were born between 37 and 42 weeks of gestation at delivery, with a minimum birth weight of 2500–4000 g, and had received a single dose of monovalent hepatitis B vaccine (Uniject™, BioFarma) at 0–7 day after birth proved by written documentation of vaccination. Infants were excluded if they had a history of allergic reaction likely to be stimulated by any vaccine component; diphtheria, tetanus, pertussis, hepatitis B, haemophilus influenzae type B infection; history of congenital or acquired immunodeficiency, uncontrolled coagulopathy or blood disorders, chronic illness, or immunosuppressive condition; or if they were undergoing immunosuppressive therapy or had received immunoglobulin therapy or blood product prior to starting or during the study; acute febrile illness at the time of the vaccination; any previous vaccination other than oral polio and BCG vaccine; and were participating in other clinical study. Infants were withdrawn from the study if after study vaccine administration they experienced fever ≥39.6 °C (axillary temperature) within 3 days of vaccination; persistent, inconsolable screaming or crying for more than 3 h within 3 days; seizures within 7 days; encephalopathy; hypotonic hyporesponsive episode within 3 days; thrombocytopenic purpura; or hypersensitivity reaction to the study vaccine.

This study was designed to evaluate the consistency of manufacturing based on immunogenicity and safety outcomes from three lots of Bio Farma DTP-HB-Hib vaccine. At the time of enrollment, subjects were assigned randomly to one of three vaccine groups in a randomized block permutation by using a randomization list.

### Study vaccine

All DTP-HB-Hib vaccines used in this study were developed and manufactured by Bio Farma, Bandung, Indonesia. Three batches of vaccines were used, batch A of which was from commercial scale, while B and C were pilot scale of production. The study vaccines were administered at 6, 10, and 14 weeks of age, with the interval between doses was 4 weeks. The study vaccines were given intramuscularly in the external anterolateral region of the thigh. All three study vaccines had the same composition. Each 0.5 ml dose contained ≥30 IU of purified diphtheria toxoid, ≥60 IU of purified tetanus toxoid, ≥4 IU of inactivated *Bordetella pertussis,* 10 μg hepatitis B surface antigen (HBsAg, recombinant), 10 μg PRP (polyribosil-ribitol-phosphate) conjugated to tetanus toxoid, 1.5 μg alumunium phosphate, 4.5 mg sodium chloride, and 0.025 mg thimerosal.

### Immunogenicity assessment

Blood samples were collected prior to the first dose of study vaccine and 28 days after the third dose to assess antibody responses. Serum samples were tested for antibodies against all vaccine antigens. Serology assays, except for anti-HBs, were conducted in Immunology Laboratory of Product Evaluation Department of Bio Farma by technicians who were blinded to group assignment. Test for anti-HBs was conducted in a commercial laboratory which had been approved by sponsor Quality Assurance.

Antibodies to tetanus and diphtheria were measured by using an enzyme-linked immunosorbent assay (ELISA). An anti-diphtheria and anti-tetanus concentration of ≥0.01 IU/ml is generally accepted to be minimum protective threshold, and a concentration of ≥0.1 IU/ml is regarded as the standard protective threshold. Pertussis antibodies were measured using microagglutination, with a cut-off set at 1/40 dilution. A vaccine response was defined as post-vaccination antibody titer four times more than the pre-vaccination titer. Antibodies to hepatitis B surface (anti-HBs) were performed using Chemiluminescent Microparticle Immunoassay (CMIA) by AUSAB, Abbott, with an assay cut-off set at 10 mIU/ml. Antibodies to PRP were measured by using Improved Phipps ELISA. A competitive Enzyme-Linked Immunosorbent Assay for measuring the levels of serum antibody to *Haemophilus influenzae* type *b* [12]. Anti-PRP concentration of ≥0.15 μg/ml is generally accepted to be minimum protective threshold, and a concentration of ≥1.0 μg/ml is regarded as the long-term protection threshold.

### Safety assessment

Safety assessments were conducted by parents and study personnel who were blinded to the three DTP-HB-Hib vaccine lots. Study personnel monitored subjects for 30 min after each vaccination to detect immediate reaction. Parents or legally guardians were given thermometers and diary cards, and were asked to record the occurrence and intensity (mild, moderate, or severe) of local (i.e. pain, redness, swelling, and induration at injection-site), and systemic (e.g. fever [≥38 °C] and irritability) reactions from day 0 (evening of vaccination) through 28 days after each vaccination. For the analyses, adverse events were graded from 1 to 3 in intensity. For local reactions, grade 3 redness, swelling, or induration was defined as areas >50 mm in diameter and grade 3 pain was defined as cried when the leg was moved. For systemic reactions, grade 3 fever was defined as axillary temperature >39 °C and grade 3 irritability was defined as inconsolable crying lasting more than 3 hours. For all other general adverse events, grade 3 was defined as preventing normal daily activities. The local and systemic reactions were classified based on the Brighton Collaboration Local Reactions Working Group and Brighton Collaboration Fever Working Group [13–15] with some modifications suggested by US Food and Drug Administration (FDA).

Parents of subjects were contacted by telephone 3 days after each vaccination to ensure completeness of reporting and to screen for adverse events (AEs) requiring medical evaluation or office visit, an emergency department visit, or hospitalization. Serious adverse events (SAEs) were recorded throughout the study and rated by investigators for possible relationship to the study vaccines. At each subsequent visit, the investigator transcribed information from the diary cards onto the Case Report Form, and asked about any other adverse experiences that occurred after the period covered by the diary card.

### Statistical analysis

The target sample size was established at 600 assessable infants for this study. A 10 % attrition rate was anticipated. Data analyses were performed using the SPSS version 18.0 (SPSS, Chicago, IL) for Windows (Microsoft Corp., Redmond, WA, USA). Demographic data were expressed as mean (SD) and range. The statistical significance of differences between the vaccine groups in demographic characteristics was assessed by Chi-square test. *P*-values <0.05 were considered to be an indicator of statistically significant difference between the vaccine groups.

The immunogenicity analyses were performed on the per-protocol population, defined as subjects who received the 3-dose primary series of the appropriately assigned study vaccines, had all blood samples obtained within the time intervals specified in the study protocol, and had a valid post-vaccination serology test result. Antibody seroprotection rates against diphtheria and tetanus toxoids, hepatitis B surface, PRP, and vaccine response rate to pertussis were calculated with 95 % confidence intervals (CI). Geometric mean antibody concentration (GMC) with 95 % CI were calculated by taking the log-transformation of individual concentration and calculating the anti-log of the mean of these transformed values. Exploratory analyses were performed to compare GMCs and seroprotection rates between the vaccine groups using Kruskal-Wallis and Chi-square or Kolmogorov-Smirnov tests. The differences of antibody concentration for each vaccine antigen before and after 3-dose primary series of DTP-HB-Hib vaccine were analyzed using Wilcoxon test.

Consistency was reached if the upper limits of the 95 % CI for the differences between groups in terms of seroprotection rates for diphtheria, tetanus, hepatitis B and PRP, and vaccine response rate for pertussis were all below the predefined limit of 10 %. With a sample size of 600 subjects, the study had at least 90 % power to conclude consistency for the co-primary objectives (α = 5 %, reference rates of 90 % seroprotection against Hib and 98 % for other parameters).

The safety analyses were based on the intention to treat population, defined as all subjects who received at least one dose of study vaccine. Exploratory analyses were performed to compare incidences of solicited local and systemic adverse events (any grade intensity) between the vaccine groups using two-sided Fisher exact test.

## Result

### Study population

**Fig. 1:**
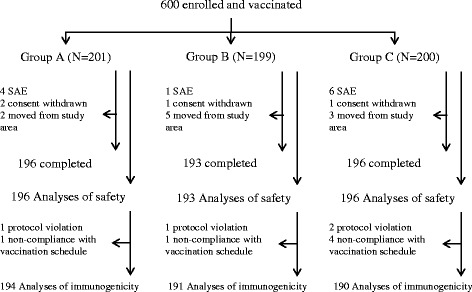
Subject disposition

A total of 600 subjects were recruited and randomly allocated to receive one of three vaccine groups, of whom 25 did not complete the study protocol: four withdrew consent; ten migrated from the study area; and one subject died due to sepsis as a bronchopneumonia complication. The remaining ten subjects were eliminated according to protocol for immunogenicity analyses: six due to non-compliance with vaccination schedule and four others due to protocol deviation for inclusion criteria (age at enrollment >11 weeks). A total of 585 infants were included in safety analyses, but only 575 infants were included in immunogenicity analyses (Fig. 1). The demographic characteristics of the subjects enrolled in each group were shown in Table 1. No clinically significant differences with respect to gender and age were observed among the three different candidate DTP-HB-Hib vaccine lots used.

**Table 1:** Summary of subject demographic characteristics (Full Analysis Set)

	Lot A	Lot B	Lot C	Total
Total, N	201	199	200	600
Gender, n (%)				
Male	114 (56.7)	110 (55.3)	97 (48.5)	321 (53.5)
Female	87 (43.3)	89 (44.7)	103 (51.5)	279 (46.5)
Age, week				
Mean ± SD	8.3 ± 1.6	8.3 ± 1.5	8.4 ± 1.6	8.3 ± 1.6
Range	6–12	6–12	5–13	5–13

### Immunogenicity assessment

Seroprotection and vaccine response rates for each antigen in the study were summarized in Table 2, and GMCs were listed in Table 3. In general, seroprotective antibody concentration for all vaccine antigens post-vaccination were no statistically significant differences between all vaccine groups. There were significant differences of antibody concentration for each vaccine antigen before and after 3-dose primary series (*p* = 0.000). Consistency of vaccines was demonstrated for all vaccine antigens. The upper limit of the 95 % CI for the difference between the vaccine groups in seroprotection or vaccine response rates was less than the predefined limit of 10 % for all antigens.

**Table 2:** Summary of seroprotection rates of antibody concentration (Per-Protocol Immunogenicity Population)

Antibody	Assessment	Criterion	Lot A			Lot B			Lot C		
			N^a^	%SP^b^	95 % CI	N	%SP	95 % CI	N	%SP	95 % CI
Diphtheria	Pre-dose 1	≥0.01 IU/ml	59	30.4	24.4–37.2	67	35.1	28.7–42.1	49	25.8	20.1–32.4
	Pre-dose 1	≥0.1 IU/ml	0	0.0	NA	7	3.7	1.8–7.4	3	1.6	0.5–4.5
	Post-dose 3	≥0.01 IU/ml	193	99.5	97.1–99.9	190	99.5	97.1–99.9	190	100.0	98.0–100.0
	Post-dose 3	≥0.1 IU/ml	168	86.6	81.1–90.7	153	80.1	73.9–85.1	162	85.3	79.5–89.6
Tetanus	Pre-dose 1	≥0.01 IU/ml	194	100.0	98.1–100.0	191	100.0	98.0–100.0	190	100.0	98.0–100.0
	Pre-dose 1	≥0.1 IU/ml	183	94.3	90.1–96.8	180	94.2	90.0–96.8	180	94.7	90.6–97.1
	Post-dose 3	≥0.01 IU/ml	194	100.0	98.1–100.0	191	100.0	98.0–100.0	190	100.0	98.0–100.0
	Post-dose 3	≥0.1 IU/ml	189	97.4	94.1–98.9	187	97.9	94.7–99.2	184	96.8	93.3–98.5
Pertussis	Pre-dose 1	≥40 (1/dil)	11	5.7	3.2–9.9	8	4.2	2.1–8.0	9	4.7	2.5–8.8
	Pre-dose 1	≥80 (1/dil)	3	1.5	0.5–4.4	3	1.6	0.5–4.5	2	1.1	0.3–3.8
	Post-dose 3	≥40 (1/dil)	172	88.7	83.4–92.4	158	82.7	76.7–87.4	161	84.7	80.6–87.1
	Post-dose 3	≥80 (1/dil)	157	80.9	74.8–85.8	140	73.3	66.6–79.1	143	75.3	68.6–81.1
	Post-dose 3	VRR^c^	173	89.2	83.9–92.9	156	81.7	77.6–84.1	159	83.7	77.7–88.4
Hepatitis B	Pre-dose 1	≥10 mIU/ml	28	14.4	10.2–20.1	33	17.3	12.6–23.3	30	15.8	11.3–21.6
	Post-dose 3	≥10 mIU/ml	191	98.5	95.6–99.5	190	99.5	97.1–99.9	190	100.0	98.0–100.0
PRP (Hib)	Pre-dose 1	≥0.15 μg/ml	57	29.4	23.4–36.1	45	23.6	18.1–30.1	57	30.0	23.9–36.9
	Pre-dose 1	≥1.0 μg/ml	22	11.3	7.6–16.6	10	5.2	2.9–9.4	19	10.0	6.5–15.1
	Post-dose 3	≥0.15 μg/ml	194	100.0	98.0–100.0	191	100.0	98.0–100.0	190	100.0	98.0–100.0
	Post-dose 3	≥1.0 μg/ml	188	96.5	93.4–98.6	183	95.8	92.0–97.9	181	95.3	91.2–97.5

NA indicates not available

^a^N = number of subjects with a valid serology result pre-dose 1 and post-dose 3

^b^%SP = seroprotection rate

^c^VRR (Vaccine Response Rate) is defined as ≥4 times more than the pre-vaccination concentration

**Table 3:** Summary of geometric mean antibody concentration (Per-Protocol Immunogenicity Population)

Antibody	Assessment	Lot A		Lot B		Lot C	
		GMC	95 % CI	GMC	95 % CI	GMC	95 % CI
Diphtheria	Pre-dose 1	0.004	0.003–0.004	0.004	0.004–0.006	0.004	0.003–0.004
	Post-dose 3	0.37	0.30–0.44	0.30	0.25–0.36	0.34	0.29–0.40
Tetanus	Pre-dose 1	1.85	1.48–2.30	1.79	1.43–2.23	1.98	1.61–2.44
	Post-dose 3	1.63	1.38–1.93	1.58	1.34–1.86	1.38	1.15–1.65
Pertussis	Pre-dose 1	6.49	5.96–7.07	6.42	5.89–7.00	6.22	5.75–6.74
	Post-dose 3	168.81	137.86–206.71	100.55	81.31–124.35	106.72	87.02–130.89
Hepatitis B	Pre-dose 1	0.005	0.003–0.008	0.007	0.004–0.01	0.006	0.003–0.01
	Post-dose 3	317.61	242.07–416.74	514.16	419.68–629.91	574.55	490.12–673.52
PRP (Hib)	Pre-dose 1	0.008	0.005–0.01	0.005	0.003–0.007	0.007	0.005–0.01
	Post-dose 3	22.13	18.32–26.73	17.72	14.50–21.65	20.32	16.50–25.03

#### Diphtheria and tetanus

After completion of 3-dose primary series, nearly all subjects in each group achieved standard protective antibody concentration (≥0.1 IU/ml) against diphtheria (86.6, 80.1 and 85.3 %, respectively) and tetanus (97.4, 97.9 and 96.8 %, respectively) toxoids (Table 2). No significant differences in GMC values (*p* = 0.337 for anti-diphtheria; and *p* = 0.479 for anti-tetanus), and seroprotection rate for concentration ≥0.01 and ≥0.1 IU/ml (*p* = 0.609 and *p* = 0.187 for anti-diphtheria; and *p* = 1.000 for anti-tetanus, respectively).

#### Pertussis

As shown in Table 2, nearly all subjects showed vaccine response rates to pertussis antigen (89.2, 81.7 and 83.7 %, respectively). Individually, GMTs were significantly higher in subjects in lot A group than in the two other groups (*p* <0.000). Although there was a significant difference in GMT, but no significant difference in the four times antibody increase result (*p* = 0.104).

#### Hepatitis B

Nearly all subjects in each group achieved seroprotective antibody concentration (≥10 mIU/ml) against hepatitis B surface antigen (98.5, 99.5 and 100.0 %, respectively) after hepatitis B vaccination at birth and 3-dose primary series (Table 2). Anti-HBs GMTs were also comparable between the vaccine groups after hepatitis B vaccination at birth and after 3-dose primary series, with a robust anti-HBs response observed after the fourth dose in all vaccine groups (Table 3). Individually, anti-HBs GMCs were significantly lower in subjects in lot A group than in the two other groups (*p* <0.001). Although there was a significant difference in GMC, but no significant difference in the four times antibody increase result (*p* = 0.859).

#### Haemophilus influenzae type b

After completion of 3-dose primary series, all subjects in each group had seroprotective anti-PRP concentration ≥0.15 μg/ml, and over 95 % from each group had concentration ≥1.0 μg/ml (Table 2). Anti-PRP GMCs pre-dose 1 and post-dose 3 were also comparable between the vaccine groups, with a robust anti-PRP response observed after the third dose in all vaccine groups (Table 3). No significant differences in GMC value (*p* = 0.174), and seroprotection rate for concentration ≥0.15 and ≥1.0 μg/ml (*p* = 1.000 and *p* = 0.704, respectively).

### Safety assessment

Each infant was counted only once and classified according to the highest grade at vaccine injection-site. Rates in lot B group were significantly lower than other group, when compared statistically by Fisher’s exact test (*p* = 0.033).

#### Immediate reactions

No anaphylactic or other severe reactions were reported to occur within 30 minutes after any dose of study vaccine.

#### Local and systemic reactions

**Fig. 2:**
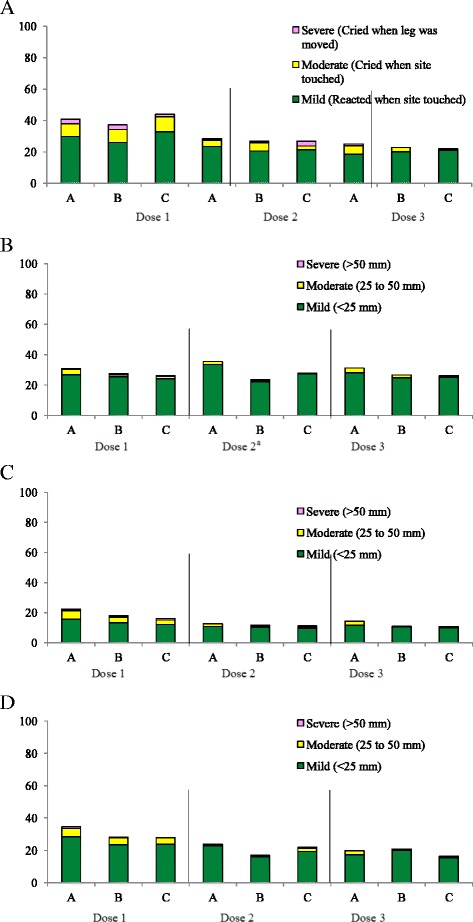
Reports of local reactions (**a**, Pain; **b**. Redness; **c**, Swelling; **d**, Induration) occurring within 72 hours after administration of DTP-HB-Hib combined vaccine

**Fig. 3:**
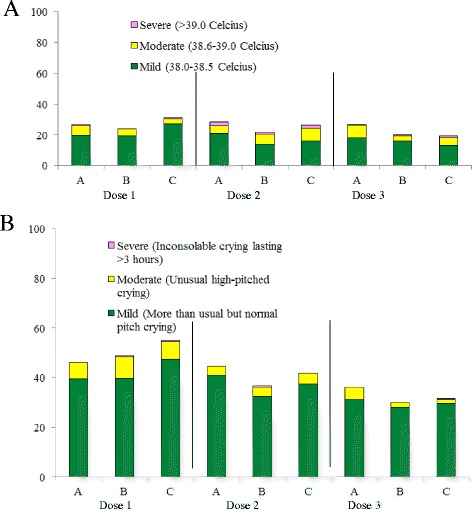
Reports of systemic reactions (**a**, Fever; **b**, Irritability) occurring within 72 hours after administration of DTP-HB-Hib combined vaccine

Figures 2 and 3 show the proportions of subjects in each group who reported the incidences of solicited local (injection-site) and systemic reactions within 72 hours after each vaccination. The most frequently reported solicited local reaction in all groups was injection-site pain (Fig. 2). The majority of local reactions in all vaccine groups were mild and resolved spontaneously within 72 hours after vaccination. Pain, swelling, and induration occurred with a similar frequency in all vaccine groups. Exploratory analyses showed that the incidence of redness was significantly higher in subjects in lot A group than the two other groups (*p* = 0.033).

The most common solicited systemic reactions were irritability (Fig. 3). Fever occurred with a similar frequency between the vaccine groups. Most of fever reactions were mild or moderate in intensity and short in duration. Severe fever (>39 °C) was reported by 0.5 % of lot A recipients, 1.0 % of lot B recipients, and 1.5 % of lot C recipients after the third dose. No hospitalizations because of fever were reported for subjects in either group. Each infant was counted only once and classified according to the highest grade.

### Serious adverse events

From the time of the first dose to 35 days after the third dose, there were 11 SAEs in 10 subjects: 3 (1.5 %) subjects in lot A group, 1 (0.5 %) in lot B group, and 6 (3.0 %) in lot C group. Seven subjects (two in lot A group, and five in lot C group) were hospitalized for 2–4 days due to acute diarrhea. Four subjects were hospitalized due to bronchopneumonia: three subjects (one in each group) recovered after treatment and one subject (lot A group) subsequently died due to sepsis. This subject from group A were hospitalized twice due to bronchopneumonia. Those SAEs were considered as coincidence and unrelated to the study vaccine and the study proceduredue to analysis from National Adverse Even Following Immunization (AEFI) committee.

## Discussion

This study analyzed the immunogenicity, safety, and consistency of the new combined DTP-HB-Hib vaccine produced by Bio Farma, when administered according the early and accelerated EPI schedule at 6, 10 and 14 weeks of age, with prior administration of a birth dose of hepatitis B vaccine, as recommended in Indonesia. The present study was conducted to generate data to support licensure of combined DTP-HB-Hib vaccine in Indonesia.

Combined vaccines have become an integral part of global childhood immunization programs and are generally highly acceptable to parents due to the relative ease of administering multiple antigens at a single visit. Multivalent vaccines have been shown to minimize the number of injection, increase compliance with the immunization schedule, increase immunization coverage, decrease exposure to vaccine excipients [16], and reduce logistic costs of vaccine delivery including number of visits to health centers, number of syringes and needles required, and necessary storage space [17, 18].

An important consideration in national immunization programs, particularly in the developing countries, is the cost effectiveness of vaccines. Gessner et al. found that for the 2007 Indonesian birth cohort, Hib vaccine would prevent meningitis in 1 of every 179 children, pneumonia in 1 of every 18 children, and 4.9 % of mortality among under-five children. The total incremental societal costs of introducing Hib vaccine in monovalent and multivalent (DTP-HB-Hib) presentations were, respectively, US$11.74 and $8.93 for each child. Annual discounted treatment costs averted amounted to 20 % of multivalent vaccine costs. For the multivalent vaccine, the incremental costs for every discounted death and disability adjusted life-year averted amounted to US$3102 and $74, respectively, versus $4438 and $102 for monovalent vaccine [19].

PRP-TT was considered to be efficacious and was approved for use in infants beginning at 6 weeks of age, without the need to perform an efficacy trial. Conceivably, the lower immunogenicity of the combination vaccines might not decrease protection among vaccinated children but could result in less-durable immunity or less-effective control of Hib colonization or transmission [20]. A further surveillance study will be required to evaluate decline incidence of Hib disease associated with the investigational DTP-HB-Hib combination vaccine.

The majority of subjects (ranging between 84 and 100 %) in each vaccine group achieved serum antibody concentration indicative of protection against diphtheria, tetanus, pertussis, hepatitis B, and Hib after 3-dose primary vaccination series. Other studies conducted in India and Philippines that used the same accelerated EPI schedule but vaccinated with different DTP-HB-Hib vaccine showed seroprotection rates similar to those observed in the present study [7, 8, 18, 21, 22].

Gatchalian et al. vaccinated 94 healthy Philippines infants with DTPw-HBV/Hib_10_ (Tritanrix™-HepB and Hiberix™, GlaxoSmithKline Biologicals, Rixensart, Belgium) with a schedule of 6, 10, and 14 weeks of age, without prior hepatitis B vaccination at birth. One month after the third dose, 100.0 and 94.7 % of subjects had anti-PRP concentration ≥0.15 and ≥1.0 μg/ml; 92.6, 100.0 and 78.5 % of subjects had seroprotective concentration against diphtheria, tetanus, and hepatitis B; and 98.9 % had pertussis vaccine response, respectively [8]. Chatterjee et al. vaccinated 89 healthy Indian infants, who had received one dose of the Hep B vaccine within 1 week of birth, with DTPw-HBV/Hib_10_ (Tritanrix™-HepB and Hiberix™) with a schedule of 6, 10, and 14 weeks of age. One month after the third dose, 100.0 % of subjects had anti-PRP concentration ≥1.0 μg/ml and seroprotective concentration against tetanus and hepatitis B; and 98.9 % of subjects had anti-diphtheria ≥0.1 IU/ml and vaccine response for anti-BPT (*Bordetella pertussis*) [22].

Of interest is the finding that in this study, transplacentally acquired antibody concentration for anti-tetanus toxoid were present in all subjects before primary vaccination series. High transplacentally acquired anti-tetanus toxoid concentration are common in Indonesia, where programs for the prevention of neonatal tetanus are implemented by vaccination to pregnant women. In addition, 30.4, 15.8 and 27.7 % of subjects had seroprotective antibody concentration against diphtheria, hepatitis B surface, and Hib before primary vaccination series. Our results indicated that the immune response to the investigational DTP-HB-Hib combination vaccine is not negatively influenced by the presence of transplacentally acquired antibody concentration. Although there was evidence of the presence transplacentally acquired antibodies at 6–11 weeks of age, the GMC values showed a marked increase after 3-dose primary vaccination series, thereby demonstrating a vaccine response in the subjects.

During the study period, the investigational DTP-HB-Hib combination vaccine elicited similar proportions of solicited local and systemic reaction between the vaccine groups. The incidence of local and systemic reactions decreased with successive doses of primary vaccination. Pain and irritability were the most frequent solicited local and systemic reactions in each vaccine group. Fewer than 3 % of local or systemic reactions were reported as severe after any dose in either group. Fever of any severity was reported at lower rates among all subjects after any dose. Fever with the first dose is of particular importance, because fever in young infants is often considered as possibly representing sepsis and thus may lead to medical and laboratory evaluation, including a visit to the physician’s office or emergency department and diagnostic testing for possible systemic infection.

In the other studies, local reactions including redness, swelling, and pain at the site of injection usually started within 1 day after vaccination and last for 1–3 days. Less commonly, children may develop a fever or be irritable for a short period. When the Hib vaccine was given at the same time as DTP, the rate of fever or irritability, or both, was no higher than when DTP was given alone [2]. In this study, the percentage of local and systemic reactions following 3-dose primary vaccination series was within the range reported for DTP-HB-based combination vaccine and licensed-equivalent vaccine [9, 21, 23–25]. Addition of each vaccine component to the DTP-HB-Hib combination kept safety profile of the investigational DTP-HB-Hib combination vaccine appeared to be similar to that of the DTP-HB-based combination vaccine and licensed-equivalent vaccines.

Lot-to-lot consistency for the investigational DTP-HB-Hib combination vaccine was demonstrated for all vaccine antigens. The upper limit of the 95 % CI for the difference between the vaccine groups in seroprotection or vaccine response rates was less than the predefined limit of 10 % for all antigens. Based on this finding, data for the three vaccine lots used in this study were pooled for comparison against each lot. This result provided empirical evidence of consistency between lot productions, which had also been verified through quality control protocols.

In Bhutan, five cases of encephalopathy and/or meningoencephalitis were reported after introduction of pentavalent vaccination in 2009. In 2012–2013, India introduced the similar vaccine resulted in 83 AEFI cases. As many as 43 serious AEFI cases including 27 fatal outcomes were also reported in Vietnam after introduction of pentavalent vaccine from Crucell in 2010–2013. All of these serious AEFI cases in each country were reviewed with independent national and international experts [11].

The safety profile of DTP-HB-Hib vaccine could be explored further in next phase. Some serious AEFIs which had not occurred in phase three study would occurred in phase four study. Hence, more accurate safety profile could be obtained for implementation of combination vaccines in the future.

## Conclusions

The investigational DTP-HB-Hib combination vaccine has proven high immunogenicity for all vaccine antigens and an acceptable safety profile. This study supports the conclusion that the Bio Farma DTP-HB-Hib combination vaccine is a suitable replacement for the licensed-equivalent vaccines based on similar safety profiles, and antibody responses to the vaccine antigens after 3-dose primary vaccination series. Replacement of standard DTP-HB vaccine, which already has high coverage, with DTP-HB-Hib can be done without modifying the existing EPI schedule. This should facilitate widespread coverage of Hib vaccination and their rapid incorporation into the EPI, and WHO recommendations for controlling Hib disease which are responsible for substantial mortality and morbidity worldwide.

## Abbreviations

DTP: diphtheria-tetanus-pertussis
HB: Hepatitis B
Hib: Haemophilus influenzae
